# Phenotypic plasticity in courtship exposed to selection in a human‐disturbed environment

**DOI:** 10.1111/eva.13225

**Published:** 2021-03-25

**Authors:** Ulrika Candolin, Irene Jensen

**Affiliations:** ^1^ Organismal and Evolutionary Biology University of Helsinki Helsinki Finland

**Keywords:** algal blooms, behaviour, environmental change, mate choice, reproduction, threespine stickleback

## Abstract

When environments change rapidly, evolutionary processes may be too slow to rescue populations from decline. Persistence then hinges on plastic adjustments of critical traits to the altered conditions. However, the degree to which species harbour the necessary plasticity and the degree to which the plasticity is exposed to selection in human‐disturbed environments are poorly known. We show that a population of the threespine stickleback (*Gasterosteus aculeatus*) harbours variation in plasticity in male courtship behaviour, which is exposed to selection when visibility deteriorates because of enhanced algal growth. Females in clear water show no preference for plastic males, while females in algal‐rich, turbid water switch their mate preference towards males with adaptive plasticity. Thus, while the plasticity is not selected for in the original clear water environment, it comes under selection in turbid water. However, much maladaptive plasticity is present in the population, probably because larger turbidity fluctuations have been rare in the past. Thus, the probability that the plasticity will improve the ability of the population to cope with human‐induced increases in turbidity—and possibly facilitate genetic adaptation—depends on its prevalence and genetic basis. In conclusion, our results show that rapid human‐induced environmental change can expose phenotypic plasticity to selection, but that much of the plasticity can be maladaptive, also when the altered conditions represent extremes of earlier encountered conditions. Thus, whether the plasticity will improve population viability remains questionable.

## 1 INTRODUCTION

Environments are currently changing at an unprecedented rate and scale because of human activities. Species with longer generation time may be unable to keep pace with the rapid changes through genetic adaptation and have to rely on phenotypically plastic adjustments of their traits—least at the initial stage of the change—to persist in the environment (Chevin & Lande, 2010; Fox, Donelson, et al., 2019). Whether populations harbour the necessary plasticity depends on the evolutionary past of the species (Sih, 2013; Tuomainen & Candolin, 2011). Species that encounter novel conditions, which they have not come across in their recent evolutionary past, may not have the plasticity needed and, hence, may face population decline and possible extinction (Ghalambor et al., 2007; Schlaepfer et al., 2002). Species that encounter conditions that are extremes of earlier encountered conditions may again possess adaptive plasticity. For instance, populations that have experienced rises in temperature or increases in precipitation in the past may have adaptive reaction norms for responding to such changes, as the changes only extend earlier encountered conditions (Chevin & Hoffmann, 2017; Kelly, 2019; Snell‐Rood et al., 2018). However, maladaptive responses can still occur, as the extreme conditions may require new responses or alterations of existing reaction norms (Chevin et al., 2010). For instance, a linear increase in activity with rising temperature may not be advantageous if inactivity is the optimal response above a certain thermal threshold to prevent the organism from overheating. In addition, the cues that individuals use to evaluate environmental conditions can become unreliable under altered conditions, which can result in maladaptive responses (Bonamour et al., 2019; Chevin & Lande, 2015; Reed et al., 2010). Thus, maladaptive responses may occur also when the environmental change only extends earlier encountered conditions.

Phenotypic plasticity influences not only the persistence of a population in the short term, but also its possibility of genetic adaptation in the long term (Fox, Donelson, et al., 2019; Kelly, 2019). Adaptive plasticity can buy time for evolutionary changes, influence the expression of genetic variation and produce new trait combinations that can be screened by selection (Crispo, 2007; Draghi & Whitlock, 2012; Kelly, 2019; Pfennig et al., 2010; West‐Eberhard, 2003). Maladaptive plasticity, on the other hand, can increase the rate of evolution by generating stronger selection against the maladaptive trait (Ghalambor et al., 2015). In addition, phenotypic plasticity itself may evolve—depending on its genetic basis—through selection on the presence and nature of the plastic responses (Chevin & Hoffmann, 2017; Kelly, 2019; Scheiner, 1993). With time, more fixed, canalized traits may evolve—if the altered conditions become the new norm—in which case the degree of plasticity may decrease (Flatt, 2005).

The presence of adaptive phenotypic plasticity in a population can consequently have both short‐ and long‐term effects on the characteristics of the population and its probability of persistence. Yet, our knowledge of the presence of phenotypic plasticity in populations, and the degree to which it is adaptive under various human‐induced environmental changes, is poor. The plasticity may not be visible before the environment changes if random factors or past conditions have selected for the plasticity, or if the phenotypic trait is constrained by pleiotrophic effects and correlations with other traits (Paaby & Rockman, 2014; Schlichting, 2008). Individuals may also show large variation in phenotypic plasticity, both in their genetically determined reaction norms—because of past variation in selection pressures and random factors—and in their expression of the plasticity, because of differences in experience during lifetime and transgenerational effects (Dingemanse & Wolf, 2013; Saltz et al., 2018; Sih, 2013; West‐Eberhard, 2003).

Sexually selected traits are often highly plastic, as they need to be adjusted to local conditions to maximize mating and fertilization success (Candolin, 2019a; Fox, Fromhage, et al., 2019). For instance, traits that attract mates have to be conspicuous and easy to evaluate under prevailing conditions, such as ornaments that need to contrast with the background, and vocalizations that have to overcome the masking effect of ambient noise. Mate preferences and mate choice should again ensure the selection of mates that are well adapted to prevailing conditions and who can provide direct and/or indirect benefits, such as high‐quality parental care or the inheritance of genes that improve offspring viability (Andersson, 1994; Endler, 1992). In addition, the costs of sexually selected traits may change with the environment, such as the energetic cost of courtship or the associated risk of predation, and, hence, require adjustments of the sexually selected traits to ensure high reproductive success (Candolin & Heuschele, 2008).

An inability to adjust sexually selected traits to changes in the environment can reduce the number and/or viability of offspring produced and, thus, the viability of the population and its likelihood of persistence. Given this importance of sexually selected traits in determining individual fitness and population viability, knowledge of the degree of plasticity in a population can be of crucial importance in predicting the dynamics of the population in a changing environment (Arnold et al., 2019; Fox, Donelson, et al., 2019; Kelly, 2019). Yet, little information exists, which restricts our ability to predict which species will be able to cope with human‐induced environmental changes and which will not, and, thus, how species communities will develop. This impedes in turn the development of effective management strategies to mitigate short‐ and long‐term negative effects of human activities.

A common human‐induced environmental disturbance, which influences the efficiency of visual sexually selected traits in aquatic environments, is the input of excess nutrients to the habitats, that is anthropogenic eutrophication (Smith et al., 2006). This promotes phytoplankton growth that reduces water clarity, which hampers the use of visual traits in mate attraction and mate choice (Alexander et al., 2017; Candolin, 2019a; Candolin et al., 2016; van der Sluijs et al., 2011). Whether aquatic species harbour the phenotypic plasticity needed to cope with the reduced visibility depends on their evolutionary history and, thus, on earlier encountered conditions. Species inhabiting areas that naturally vary in turbidity could harbour the necessary adaptive plasticity, which could ensure their persistence in the short term, and possible in the longer term by providing more time for genetic adaptation and by exposing genetic variation to selection (Chevin et al., 2010; Fox, Donelson, et al., 2019; Kelly, 2019).

We used a population of the threespine stickleback (*Gasterosteus aculeatus*) in the Baltic Sea to investigate: (1) if the population harbours phenotypic plasticity in male courtship behaviour that is not evident under the natural clear water conditions, (2) if the plasticity is exposed to selection when turbidity increases because of microalgae growth and (3) if the plasticity is adaptive and under selection when turbidity increases. The population has experienced variation in turbidity levels in the past (Andersen et al., 2017) and, thus, could harbour adaptive plasticity for copying with turbidity increases. Turbidity levels vary in the spawning habitats of the species—shallow coastal waters—because of temperature fluctuations and exchange of nutrients and phytoplankton with the open sea. Turbidity levels increase under favourable conditions and decrease when nutrients have been used up, temperature drops, water exchange increases, or consumer density grows (Carstensen et al., 2015; O'Neil et al., 2012).

Recent modelling predicts that turbidity levels will increase in the Baltic Sea in the near future because of interactions between eutrophication and global warming (Meier et al., 2019). This could influence the adaptedness of the mating behaviour of the threespine stickleback, as males use visual signals to attract females to spawn in a nest they have built, that is they perform a conspicuous courtship dance that is combined with bright nuptial coloration (Candolin et al., 2007; Engström‐Öst & Candolin, 2007). Earlier research shows that algal turbidity hampers the ability of females to evaluate courting males and make adaptive mate choices (Candolin, 2009; Candolin et al., 2016). However, the degree to which males can adjust their courtship behaviours to improve visibility under turbidity increases is unknown. Knowledge of the capacity to adjust would improve our ability to understand and predict changes in the population dynamics of the species. Given the central role that the threespine stickleback plays in the ecosystem, as a dominant mesopredator that influences the dynamics of a range of other species and ecological processes (Candolin, 2019b), this could improve our ability to understand and predict also other changes in the ecosystem.

## 2 METHODS

### 2.1 Maintenance

We caught threespine stickleback before the breeding season from a bay in the outer archipelago of the Northern Baltic Proper (60°N, 23°E) using Plexiglas traps (Candolin & Voigt, 2001). The bay has clear water, ~1 nephelometric turbidity unit (NTU). We housed the stickleback in large holding tanks at a density of 0.25 fish per litre, a temperature of 18°C, a photoperiod of 18L:6D and a salinity of 5.5 psu. We fed the fish defrosted chironomid larvae once a day. When males came into reproductive condition, as determined by the development of nuptial colouration, we moved them to individual 10‐L tanks. Each tank contained an artificial plant for hiding and material for nest building: a nesting dish (Ø 12.5 cm) filled with sand and filamentous algae (see Candolin, 1997). To stimulate nest building, we presented the males with a gravid female, enclosed in a transparent, perforated, plastic cup, for 15 min twice a day. In the experiment, we used only males that built a nest within 2 weeks.

### 2.2 Experimental tanks

To investigate whether increases in water turbidity exposes phenotypic plasticity in male courtship behaviour, we allowed males to court females in both clear and turbid water, alternating the order among males. We moved males with completed nests, together with their nesting dishes, to experimental flow‐through tanks (70 × 30 cm, water height 15 cm), one male into each tank. Males were moved in the afternoon before courtship recordings to give them time to acclimatize to the experimental tank. The tanks contained artificial vegetation evenly distributed over the bottom so that about 50% of the bottom was covered by bunches of 10 cm long, thin polypropylene strings (Candolin et al., 2007). The vegetation density corresponded to natural conditions in the field (Candolin, 2004).

To manipulate the level of turbidity, we either left the water in the tank as clear water (~1 NTU) or gradually increased turbidity to ~15 NTU by adding the nontoxic flagellate algae *Isochrysis* sp. to the inflow water. The clear water treatment reflected water conditions in the spawning habitat from which the stickleback had been caught, and the turbidity treatment conditions in the inner part of the archipelago, where turbidity can reach 15 NTU during algal blooms (Salonen et al., 2009). Thus, the turbidity treatment reflected levels that could be recorded in the future in the outer part of the archipelago if algal growth intensifies as expected (Meier et al., 2019). The selected algae is a common species in the Baltic Sea. We cultivated the algae according to previously published methods (Vlieger & Candolin, 2009). The inflow rate of the water with the algae was 150–200 ml min^−1^, and we measured turbidity level in the tank with a portable nephelometer (Hach 2100P). When the sought turbidity level had been attained, we stopped the water flow in both the clear and turbid water treatments. Airstones kept the water in the tanks constantly aerated.

### 2.3 Behavioural observations

The following morning, we checked the turbidity level in the tank and adjusted it when needed, ensuring that both treatments experienced the same level of disturbance by disturbing the water also in tanks where no adjustment was needed. When the right turbidity level had been attained, an observer recorded the mate search behaviour of the male from behind a blind. Four different observers did the observations, who had been thoroughly instructed before the start of the experiment. The identity of the observers did not influence the results and the observer effect term was deleted from the final models in the analyses. To record mate search behaviour, we had marked four 16 cm wide zones at the bottom of the tank, starting from the centre of the nesting dish. During 15 min, we recorded the amount of time the male spent within each zone. To gain a measure of search activity, we multiplied the time spent in each zone with the distance to the nest (with the zone furthest from the nest given a distance value of 4) and summed the values for the four zones. Thus, the measure considers both time spent away from the nest and the distance to the nest, and higher values indicate higher search activity.

After mate search recording, the observer recorded the courtship behaviour of the male by placing a gravid female, enclosed in a transparent, perforated cylinder (Ø 12 cm), 50–60 cm from the nest. The male was by his nest when the female was added, and the vegetation in the tank prevented him from observing the introduction of the female. During 10 min, the observer recorded the time elapsed until the male noticed the female by orienting towards her, the number of leads towards the nest (the male moves in a straight line towards the nest and then returns to the female), the number of fanning bouts at the nest entrance (the male fans fresh water into the nest with his pectoral fins), the total time spent fanning, the total time spent by the female (within 5 cm of the cylinder enclosing the female) and the total time spent by the nest.

After the 10 min of courtship recording, we released the female and noted whether she inspected the nest of the male within 15 min. We prevented the female from spawning if she showed indications of entering the nest, by blocking the nest entrance with a long transparent stick. We removed the female immediately after nest inspection, or at the end of the 15 min if no nest inspection occurred. Females that do not inspect a nest within 15 min are usually not interested in spawning with a male (U. Candolin, personal observation). We repeated the procedures with two additional females, with a 1 h break between female presentations. Before each female presentation, we recorded the search behaviour of the male for 15 min, as detailed above.

After the three female presentations, we gradually altered the turbidity of the water to the opposite treatment. The following day, we repeated the procedures from the first treatment by recording male search activity, courtship behaviour, and female interest in spawning with the males. We tested 42 males, with the order of the turbidity treatments alternated among replicates. The order term was included in the models in the analyses, but deleted as no significant effects were found. All females were used only once, and they were randomly allocated to males.

### 2.4 Analyses

The recorded male behaviours were correlated (Table S1), and we used two approaches to analyse the data: (1) basing the analyses on the three main behaviours recorded: search activity, time spent by the female, and time spent by the nest (during courtship recording) and (2) calculating principal components (PCs) of all recorded behaviours, which were standardized before calculation. The former approach allowed us to separate between the relative impact of the three main behaviours on female willingness to spawn, while the latter allowed us to determine the general effect of male courtship activity.

To analyse whether water turbidity exposed or hid variation among males in courtship activity, we compared their variation in activity between clear and turbid water. We used the Pitman–Morgan test for homogeneity of variances for paired variables, as the same males were tested in both treatments (Morgan, 1939; Pitman, 1939). We used the equation in Gardner (2001, p 57) and based it on the mean of the three recorded behaviours during the three female presentations, and on the mean PC scores (see Equation S1).

To analyse whether female willingness to inspect a male depended on his plasticity in courtship activity between clear and turbid water, we calculated an index of plasticity for each male. It was based on the mean values of the recorded behaviours with the three females, that is the difference in activity between clear and turbid water. The index was calculated for the three separate behaviours as: (behaviour in turbid − behaviour in clear)/(behaviour in turbid + behaviour in clear). For the principal component scores, the index was calculated as the difference between the scores in clear and turbid water. To analyse female preference for plastic males, we used general linear mixed models (GLMMs) with binomial error distribution and a log link. The analyses were performed separately for females in clear and turbid water, and by combining the two treatments to investigate whether the patterns differed between treatments. The response variable was the bivariate nest inspection decision, fixed factors were the calculated plasticity indexes, and the random factor was male identity.

To analyse whether females preferred males that were consistent (fixed) in their behaviours within each treatment, we calculated the coefficients of variation (CV) for the three behaviours and for the PC scores. We used similar GLMMs for analysing female preference for plastic males, with the coefficients of variation within each treatment as fixed factor.

To analyse whether females preferred the same males in clear and turbid water, we summed the number of females that inspected the nest of a male (0–3) and used the nonparametric Spearman's rank correlation to test for concordance between treatments.

To analyse whether female interest in the males differed between treatments, we used the nonparametric Wilcoxon signed‐rank test for two related samples to test for differences in the number of females that inspected each male.

## 3 RESULTS

The first principal component (PC1) from the principal component analysis of the recorded male behaviours explained 46% of the variation (Tables S2 and S3). It reflected the three main behaviours: search activity, time by the female and time by the nest. Larger positive values reflected more activities further from the nest, that is higher search activity, more time spent by the female (including leads between the female and the nest) and less time spent by the nest (see Supporting Information). The second principal component (PC2) explained 32% of the variation and reflected fanning activity at the nest (Tables S2 and S3).

Turbid water increased the variation among males in the three main behaviours and in PC1 compared with clear water, that is in search activity, time spent by the female and time spent by the nest (Table 1). The variation in PC2 did not differ between treatments (Table 1) and is not further considered in the analyses.

**TABLE 1:** The variation (SD) among threespine stickleback males in courtship activity in clear and turbid water. Differences between treatments were analysed using the Pitman–Morgan test for homogeneity of variances between two dependent variables

	Clear	Turbid	*t*_40_	*p*
Search activity	62.6	184.9	3.84	<0.001
Time by female	85.8	196.6	2.80	0.008
Time by nest	54.9	201.9	5.06	<0.001
PC1	0.344	1.269	5.72	<0.001
PC2	0.503	0.512	0.06	0.955

**FIGURE 1:**
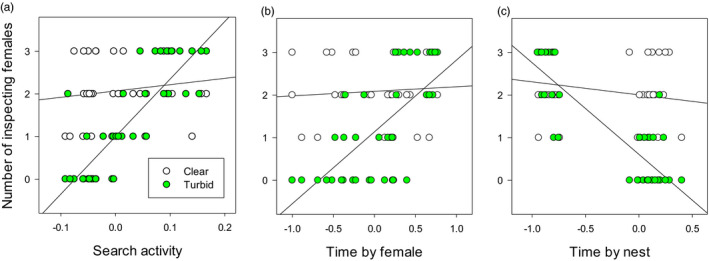
Number of threespine stickleback females that inspected the nest of a male in clear and turbid water depending on the plasticity of the male in (a) search activity in clear water (*b* = 1.48, SE = 1.47) and in turbid water (*b* = 13.50, SE = 1.34), (b) time spent by the female in clear water (*b* = 0.11, SE = 0.23) and in turbid water (*b* = 1.69, SE = 0.29), and (c) time spend by the nest in clear water (*b* = −0.30, SE = 0.23) and in turbid water (*b* = −2.15, SE = 0.19). Higher values indicate higher plasticity in the behaviours between clear and turbid water, and positive values indicate higher activity in turbid water

Females showed no preference for plastic males in clear water (i.e. males that varied their courtship activity between clear and turbid water), neither when analysing the three individual behaviours separately, nor when considering correlations between the behaviours using a multivariate model, or using the PC1 as the dependent variable (Table 2, Figure 1). Females in turbid water, on the other hand, preferred plastic males who increased their search activity and time spent by the female, while decreasing the time spent by the nest (Table 2, Figure 1). The patterns differed significantly between treatments (interaction terms in Table 2). Multivariate models revealed that the strongest effect on female interest came from increased search activity and reduced time spent by the nest, that is behaviours that increased visibility to the female (Table 2).

**TABLE 2:** The dependence of nest inspection decision of threespine stickleback females on male courtship plasticity in search activity, time spent by the female, time spent by the nest and the first principal component score of all recorded behaviours. Plasticity is measured as difference in behaviour between clear and turbid water. Results are presented for the two treatments, and for the interaction between treatment and male plasticity. Both univariate and multivariate GLMMs with binomial error distribution were run. In the multivariate model, only significant interactions are kept. All random effect parameters, male identity, were nonsignificant

	Univariate				Multivariate			
	Coeff	SE	*F*_1,124_	*p*	Coeff	SE	*F*_1,122_	*p*
Female nest inspection in clear water								
Search activity	2.39	2.63	0.83	0.363	0.36	4.01	0.01	0.930
Time by female	0.17	0.40	0.18	0.674	−0.29	0.54	0.29	0.592
Time by nest	−0.49	0.41	1.46	0.229	−0.65	0.69	0.89	0.347
PC1	0.09	0.18	0.24	0.623				
Female nest inspection in turbid water								
Search activity	26.34	4.25	38.42	<0.001	14.13	4.88	8.40	0.004
Time by female	3.61	0.79	21.02	<0.001	0.66	0.79	0.69	0.407
Time by nest	−3.76	0.55	47.12	<0.001	−2.02	0.75	7.22	0.008
PC1	1.97	0.30	43.48	<0.001				

	Univariate				Multivariate			
	Coeff	SE	*F*_1,248_	*p*	Coeff	SE	*F*_1,245_	*p*
Interaction between water treatment and male plasticity on female nest inspection								
Search*Treatment	23.96	4.99	23.06	<0.001				
Time female*Treatment	3.31	0.78	18.07	<0.001	15.13	6.15	6.05	0.015
Time nest*Treatment	3.27	0.68	22.98	<0.001	−1.83	0.91	4.088	0.044
PC1*Treatment	1.88	0.35	15.04	<0.001				

Abbreviation: GLMM, general linear mixed model.

The proportion of males that altered their behaviour in the direction preferred by females in turbid water—who showed adaptive plasticity—was 45% (19/42) for search activity, 38% (16/42) for time spent by the female, and 50% (21/42) for time spent by the nest (see Figure S1). Only 21% of the males (9/42) changed all three behaviours in the direction preferred by females.

Females in clear water showed no preference for males with high consistency in courtship behaviour (across the three female presentations) in clear or in turbid water (Table 3). Females in turbid water, on the other hand, preferred males that were consistent in their behaviour in turbid water, especially in search activity and time spent by the nest (Table 3). Consistency in clear water had no significant impact on female interest in turbid water (Table 3).

**TABLE 3:** The dependence of nest inspection decision of female threespine stickleback on male consistency in search activity, time spent by the female, time spent by the nest and the first principal component score of all recorded behaviours in clear and in turbid water. Consistency is measured as coefficient of variation (CV) in the behaviours towards three females. Results are from univariate and multivariate GLMM with binomial error distribution. Variance estimates for the random factors, male identity, are shown when these are significant (*p* < 0.05)

	Univariate						Multivariate					
	Fixed effects				Random effect		Fixed effects				Random effect	
	Coeff	SE	*F*_1,124_	*p*	Estimate	SE	Coeff	SE	*F*_1,122_	*p*	Estimate	SE
Female nest inspection in clear water												
Male consistency in clear water												
Search activity	0.323	0.47	0.01	0.923			0.06	3.37	0.01	0.986		
Time by female	−0.56	0.81	0.47	0.495			−0.44	0.90	0.23	0.630		
Time by nest	−0.80	1.37	0.34	0.563			−0.48	1.53	0.10	0.755		
PC1	−0.02	0.22	0.56	0.454								
Male consistency in turbid water												
Search activity	−5.60	5.42	1.07	0.304			−8.76	7.26	1.45	0.230		
Time by female	0.69	0.56	1.52	0.219			0.71	0.64	1.24	0.268		
Time by nest	0.23	0.73	0.10	0.749			1.32	0.95	1.94	0.166		
PC1	−0.31	0.40	0.60	0.440								
Female nest inspection in turbid water												
Male consistency in clear water												
Search activity	−5.27	5.59	0.89	0.348	2.46	1.03	−5.67	5.73	0.98	0.324		
Time by female	−0.20	0.59	1.64	0.203	2.52	1.04	2.64	1.55	2.92	0.090		
Time by nest	−1.12	2.15	0.27	0.602	2.46	2.39	−3.39	2.51	1.82	0.179		
PC1	−0.01	0.03	0.21	0.649	2.52	1.04					2.58	1.10
Male consistency in turbid water												
Search activity	2.32	0.49	22.35	<0.001			−22.60	7.62	8.80	0.004		
Time by female	3.64	1.12	10.61	0.001	1.81	0.87	1.77	0.90	3.85	0.052		
Time by nest	−6.69	1.54	18.88	0.000			−3.93	0.64	7.45	0.007		
PC1	−1.17	0.64	3.38	0.068	2.25	0.98						

Abbreviation: GLMM, general linear mixed model.

Female mate preference changed between treatments, as the males that were preferred in clear and turbid water did not align (Spearman's Rank Correlation coefficient = 0.14, *N* = 42, *p* = 0.38). In general, females were less interested in the males in turbid water, as fewer females inspected the nest of a male: median number of inspecting females was two out of three in clear water (mean = 2.10), and one out of three in turbid water (mean = 1.24), Wilcoxon signed‐rank test, *N* = 42, *Z* = 87, *p* < 0.001).

## 4 DISCUSSION

Our results show that increased algal turbidity exposes variation in plasticity in courtship behaviour to selection in the threespine stickleback and that this strengthens selection on adaptive plasticity. Females in turbid water preferred males that increased activities that improved their visibility to females, that is males who increased their search activity and time spent by the female, and decreased the time spent by the nest. Stickleback females inspect a male at a distance before deciding whether to follow him to his nest and, hence, may not be able to detect a male, or evaluate his quality, in turbid water if he spends most of his time by the nest engaged in nest‐oriented behaviours. In clear water, on the other hand, nest‐oriented behaviours attract females, probably because they reflect the parenting ability of the male (Candolin et al., 2007). Thus, males benefit from switching from activities by the nest to activities further from the nest when water turbidity increases.

Interestingly, much maladaptive plasticity was present in the population. The majority of the males adjusted their behaviours in the wrong direction when turbidity increased, by spending more time by the nest rather than further from it. The cause of the high prevalence of maladaptive plasticity could be opposing selection or weak past selection for adaptive plasticity (Murren et al., 2015). Regarding opposing selection, intense male–male competition is likely to have selected for the behaviour to stay close to the nest, to protect it against raiding males. In addition, costs of venturing further from the nest could have selected against adaptive plasticity, such as an increased risk of encountering predators or parasites, risk of fights with neighbouring males or increased energy expenditure. Increased predation risk is unlikely to explain the lack of adaptive plasticity, as courting males should be less visible to predators in turbid water and poor visibility weakens rather than heightens antipredator responses (Candolin, 1997; Candolin & Voigt, 1998; Johnson & Candolin, 2017; Sohel & Lindström, 2015). However, males could have responded with neophobia to higher turbidity levels, staying close to their nest because of increased uncertainty about local risks (Mettke‐Hofmann, 2014; Schmidt et al., 2010). Threespine stickleback show large individual variation in behaviours such as boldness and general activity (Bell & Sih, 2007; Candolin & Voigt, 1998; Dingemanse et al., 2007; Sohel & Lindström, 2015), and the large variation in plasticity could be caused by indirect selection on these behaviours rather than by direct selection on responses to turbidity.

The lack of past selection for adaptive plasticity could explain the high prevalence of maladaptive plasticity in the population, as the investigated population spawns in clear water and has not experienced high turbidity levels in the past, as these are more common in inshore areas (Salonen et al., 2009). In support of this, females in clear water showed no preference for or against males with adaptive plasticity. Lack of past selection could also explain the large variation among males in responses to turbidity.

While high turbidity levels selected for plasticity between clear and turbid water, it selected for consistency in behaviour under turbid water conditions; females in turbid water preferred males that were consistent in their courtship behaviour under turbid water conditions, although not necessarily under clear water conditions. In clear water, on the other hand, consistency in male behaviour had no impact on female nest inspection, neither consistency under clear water conditions nor under turbid water conditions. The cause of the preference for males that are consistent in their behaviour in turbid water could be a restriction in the number of cues that females can evaluate in turbid water, or a more limited range of values that attract females, resulting in only particular trait values being favoured. In particular, a lack of past exposure to turbid water could have prevented the evolution of efficient mate evaluation behaviour in turbid water. Females in clear water pay attention to several visual traits, such as nuptial coloration, body size, and body symmetry (Kunzler & Bakker, 2001), but these traits are difficult to evaluate in turbid water (Heuschele et al., 2009). Moreover, the variation among females in mate preferences could have been lower in turbid water if females adjusted their mate preferences less to their own characteristics under turbid conditions (Bakker et al., 1999). Alternatively, males may have been less prone to adjust their courtship behaviour to the characteristics of the females, as visual traits of females should be more difficult to evaluate in turbid water, resulting in a lower variation within males in courtship behaviour.

The result that different males were preferred in clear and turbid water indicates that turbid water alters selection acting on the population. This could influence not only the evolution of plasticity and the investigated traits but also traits correlated with courtship, or exposed to selection through courtship (Hendry, 2016; West‐Eberhard, 2003). For instance, red nuptial coloration and morphological traits are exposed to selection through courtship (Candolin et al., 2007), and different personality traits are correlated with general activity (Schuett et al., 2010). Thus, plasticity could expose or hide genetic variation in a suit of traits and, hence, influence the evolutionary trajectory of the species (Fox, Donelson, et al., 2019). Whether this would result in an evolutionary response and alter the composition of the population depends on the strength of selection, the underlying genetic basis of the traits and other selection pressures acting on the population (Arnold et al., 2019). Courtship behaviour has a genetic basis and has differentiated among populations, which indicates that it could evolve in response to the altered selection (Boughman et al., 2005; Foster, 1995; Foster et al., 2008; Tuomainen et al., 2011). However, other environmental perturbations could alter the ultimate selection pressure on the population, such as climate change, and thereby the evolutionary trajectory of the species (Abram et al., 2017; Crozier & Hutchings, 2014; Rosenthal & Elias, 2019). The ultimate impact on the population depends also on how turbidity levels develop in the future. If the frequency or intensity of turbidity fluctuations increase, selection could favour a high level of plasticity, while constantly high turbidity levels could favour the evolution of more rigid courtship behaviour.

Whether the exposure of plasticity to selection will influence the viability of the population depends in turn on the impact of the plasticity on female fitness. Male courtship behaviour in clear water reveals fitness benefits to females, such as parenting ability and the inheritance of genes that improve offspring viability (Candolin et al., 2016). Turbid water reduces the reliability of these behaviours as indicators of the benefits (Candolin et al., 2016), as well as the ability of females to assess these traits (Heuschele et al., 2009; Wong et al., 2007). The plasticity detected in this study could facilitate mate evaluation, but the recorded high prevalence of maladaptive plasticity reduces the likelihood. A further, unexplored possibility is that females harbour adaptive plasticity in mate preferences that becomes exposed to selection when turbidity increases, which could promote the evolution of a better‐adapted mating system.

In general, phenotypic plasticity has been proposed to influence the acclimatization and adaptation of species to human‐induced rapid environment changes (Chevin et al., 2010). Plasticity can provide a fast response, but—as shown in this study—may not necessarily be in the direction favoured by selection. Thus, plasticity can hinder as well as facilitate adjustment to altered conditions (Ghalambor et al., 2007). In the longer term, the exposure of plasticity to selection could promote evolutionary changes, depending on its genetic basis, and promote adaptation to the altered conditions (Kelly, 2019; Lande, 2009), presuming that the population survives the initial stages of environmental change.

To conclude, our results show that a human‐induced environmental change—increased algal turbidity—exposes variation in plasticity in courtship behaviour to selection in a threespine stickleback population. However, much maladaptive plasticity is present in the population, probably because of the lack of past selection for adaptive plasticity. Thus, the ultimate impact of the plasticity on the population is unclear. Turbidity levels are expected to increase in the future if eutrophication and global warming continue (Meier et al., 2019). Males with adaptive plasticity may then become under selection and their presence in the population increase and promote population persistence. However, this depends on their frequency in the population and the genetic underpinnings of the plasticity. At a more general level, our results emphasize the importance of investigating the presence of plasticity not evident under natural, undisturbed conditions and its adaptive value when evaluating the ability of species to cope with rapid human‐induced environmental changes.

## CONFLICT OF INTEREST

The authors declare that they have no conflict of interest.

## Supporting information

Supplementary Material

Supplementary Material

## Data Availability

Data for this study are available in the Supporting Information.
