# Supplementary material for: Phenotypic plasticity in courtship exposed to selection in a human‐disturbed environment
Source: Evol Appl. 2021 Mar 25;14(10):2392–401. doi: 10.1111/eva.13225 (PMC8549619; doi:10.1111/eva.13225)
Supplement: Supplementary file 1 — Supplementary Material [file EVA-14-2392-s001.pdf]

Supplementary material

Phenotypic plasticity in courtship exposed to selection in a human-disturbed environment

Ulrika Candolin and Irene Jensen

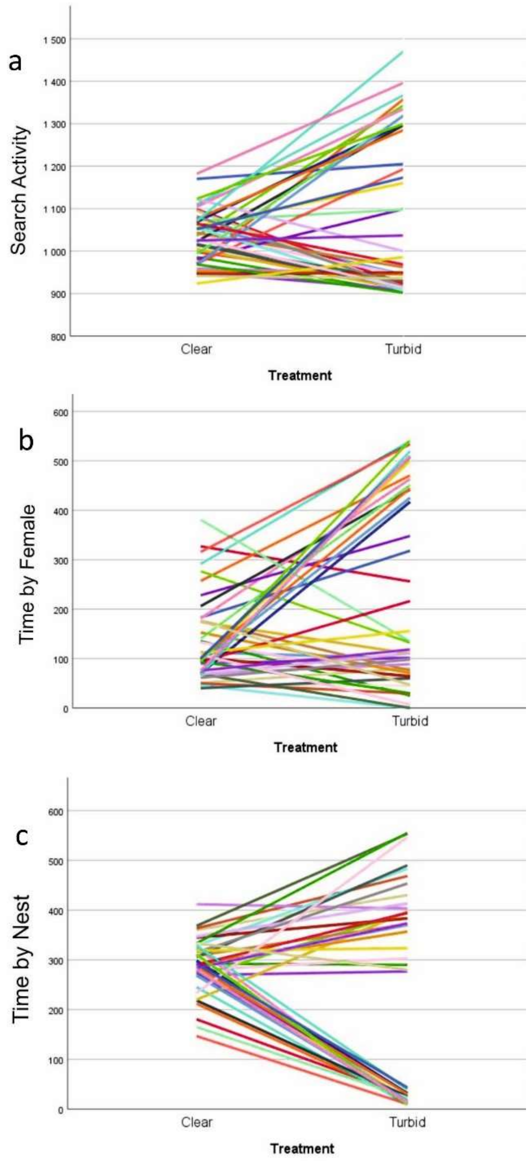

Figure S1. Reaction norms for males when water quality changes between clear and turbid, for a) search activity, b) time in seconds spent by the female during courtship, and C) time in seconds spent by the nest during courtship.

Equation 1. The Pitman-Morgan Equation used to calculate the t-value used to test for homogeneity of variances for paired variables.

$$T = \frac{\sqrt{(n-2)}[(SD_1/SD_2)-(SD_2/SD_1)]}{2\sqrt{(1-r^2)}}$$

Table S1. Correlation coefficient of the behaviours included in the principal component analysis

Pearson correlations

	Search Activity	Time to noticed female	Number leads	Number fanning bouts	Time fanning	Time by female	Time by nest
Search Activity	1	-,364**	,504**	-,033	-,083	,611**	-,570**
Time until noticed female	-,364**	1	-,402**	-,261**	-,229**	-,410**	,518**
Number of leads	,504**	-,402**	1	,534**	,413**	,560**	-,591**
Number of fanning bouts	-,033	-,261**	,534**	1	,933**	-,154*	,010
Time fanning	-,083	-,229**	,413**	,933**	1	-,217**	,105
Time by female	,611**	-,410**	,560**	-,154*	-,217**	1	-,831**
Time by nest	-,570**	,518**	-,591**	,010	,105	-,831**	1

**. Correlation is significant at the 0.01 level (2-tailed).

*. Correlation is significant at the 0.05 level (2-tailed).

Table S2. Results from a principal component analysis including all recorded male behaviours: search activity, time elapsed until noticed the female, number of leads towards the nest, number of fanning bouts at the nest entrance, total time spent fanning, total time spent by the female, and total time spent by the nest.

Total Variance Explained

Component	Initial Eigenvalues			Extraction Sums of Squared Loadings		
	Total	% of Variance	Cumulative %	Total	% of Variance	Cumulative %
PC 1	3,223	46,042	46,042	3,223	46,042	46,042
PC 2	2,243	32,043	78,085	2,243	32,043	78,085
PC 3	,660	9,427	87,512			
PC 4	,478	6,832	94,344			
PC 5	,192	2,739	97,083			
PC 6	,154	2,194	99,277			
PC 7	,051	,723	100,000			

Extraction Method: Principal Component Analysis.

Table S3. Loadings of each recorded behaviour to the principal components (PC) 1 and 2.

Component Matrix

Behaviour	PC 1	PC 2
Search Activity	,734	-,272
Time until noticed the female	-,677	-,128
Number of leads to the nest	,831	,314
Number of fanning bouts at the nest	,291	,937
Time spent fanning	,198	,950
Total time spent by the female	,818	-,431
Total time spent by the nest	-,862	,297

Extraction Method: Principal Component Analysis.
